# Synthetic non-classical luminescence generation by enhanced silica nanophotonics based on nano-bio-FRET

**DOI:** 10.1039/d0ra02939d

**Published:** 2020-05-29

**Authors:** Carina Salinas, María Valeria Amé, A. Guillermo Bracamonte

**Affiliations:** Instituto de Investigaciones en Físico Química de Córdoba (INFIQC), Departamento de Química Orgánica, Facultad de Ciencias Químicas, Universidad Nacional de Córdoba, Ciudad Universitaria 5000 Córdoba Argentina gbracamonte@fcq.unc.edu.ar; Centro de Investigaciones en Bioquímica Clínica e Inmunología (CIBICI), Departamento de Bioquímica Clinica, Facultad de Ciencias Químicas, UNC Argentina; Departement de Chimie, Centre d'Optique, Photonique et Laser (COPL), Université Laval Québec (QC) G1V 0A6 Canada

## Abstract

Fluorescent silica nanoparticles (NPs–(SiO_2_–Fluo)) were synthesized based on the classical Störber method for cyanobacteria labelling. Modified mono-coloured SiO_2_ NPs with fluorescein (Fl) and rhodamine B (RhB) were obtained (NPs–(SiO_2_–Fl) and NPs–(SiO_2_–RhB)). Moreover, multi-coloured SiO_2_ NPs, *via* the incorporation of both emitters (NPs–(SiO_2_–RhB–Fl)), were tuned for optimal emissions and the biodetection of cyanobacteria. NPs–(SiO_2_–Fl) and NPs–(SiO_2_–RhB–Fl) were optimized for detection *via* laser fluorescence microscopy and in-flow cytometry with laser excitation and fluorescence detection. By TEM, homogeneous SiO_2_ NPs of 180.0 nm in diameter were recorded. These sizes were slightly increased due to the covalent linking incorporation of fluorescent dye emitters to 210.0 nm with mono-coloured fluorescent modified amine-organosilanes, and to 340.0 nm in diameter with multi-coloured dye incorporation. NPs–(SiO_2_–Fluo) showed variable emission depending on the dye emitter concentration, quantum yield and applied luminescent pathway. Thus, mono-coloured NPs–(SiO_2_–Fl) and NPs–(SiO_2_–RhB) showed diminished emissions in comparison to multi-coloured NPs–(SiO_2_–RhB–Fl). This enhancement was explained by fluorescence resonance energy transfer (FRET) between Fl as a fluorescent energy donor and RhB as an energy acceptor produced within the nanoarchitecture, produced only in the presence of both fluorophores with the appropriate laser excitation of the energy donor. The depositions of the nano-emitters on cyanobacteria by non-covalent interactions were observed by TEM and laser fluorescence microscopy. For multi-coloured NPs–(SiO_2_–RhB–Fl) labelling, bio-FRET was observed between the emission of the nano-labellers and the natural fluorophores from the cyanobacteria that quenched the emission of the whole nano-biostructure in comparison to mono-coloured NPs–(SiO_2_–Fl) labelling. This fact was explained and discussed in terms of different fluorescence energy transfer from the nanolabellers towards different natural chromophore coupling. In the presence of NPs–(SiO_2_–RhB–Fl) and NPs–(SiO_2_–RhB), the emission was coupled with lower quantum yield chromophores; while upon the application of NPs–(SiO_2_–Fl), it was coupled with higher quantum yield chromophores. In this manner, for enhanced luminescent nanoplatform tracking, the multi-coloured NPs–(SiO_2_–RhB–Fl) showed improved properties; but more highly luminescent bio-surfaces were generated with mono-coloured NPs–(SiO_2_–Fl) that permitted faster cyanobacteria detection and counting by laser fluorescence microscopy, and by in-flow cytometry with laser excitation and fluorescence detection.

## Abstract 2

Fluorescent silica nanophotonics for cyanobacteria labelling.

## Graphical abstract

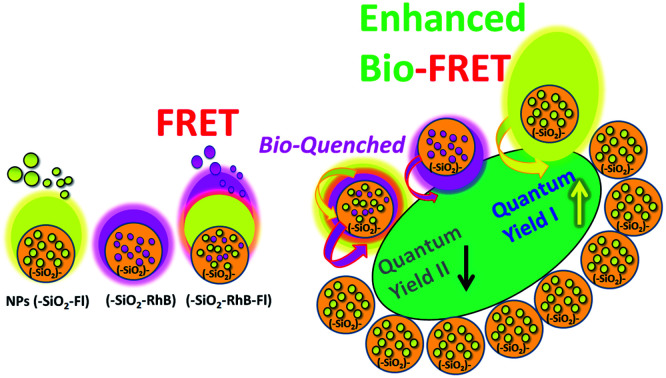

## 1. Introduction

Bacterial detection has been shown to be a high-impact research field within biology, biochemistry and clinical chemistry that is still a challenge due to needs related to biodetection coupled to higher level of information collected from individual biostructures. In this manner the signal collected should transduce from the total biostructure surface to arrive at molecular event detection within the biostructure. For these reasons the advent of bioimaging, by different techniques and chemical approaches, is in progress with high impact on new advanced instrumentation and new products on the market available for researchers as well as to professionals from different fields. In addition new phenomena from physics coupled to chemical properties permit new research studies and developments.

In order to realize bioimaging, it could be applied different approaches, but it should be mentioned that fluorescence due to its intrinsic high sensitivity is widely used and well developed. However, it is still a challenge for targeted enhanced applications.

From the literature it could be mentioned tuning *in vivo* Gram positive and negative bacteria with red fluorescent dyes for imaging, at 650 nm emission wavelengths,^1^ where it was studied and overcame many aspects related to the specific incorporation of organic molecules within membranes and background signalling. Moreover, other strategies as applications of different luminescent nanoarchitectures should be highlighted. Examples are self-assembled quantum dots as fluorescence resonance energy transfer (FRET) donors in the presence of fluorescent modified saccharide membranes of *Escherichia coli* bacteria.^2^ Others are fluorescence energy transfer inhibition bioassays for cholera toxin based on galactose-stabilized gold nanoparticles and amine-terminated quantum dots.^3^ In these examples, it was highlighted the importance of the main role of the control of energy transfer by light stimulation for biodetection applications. In addition studies related to high-energy electromagnetic fields generated from metallic surfaces within the near field at the nanoscale and their interactions with fluorescent molecules showed enhanced emissions named as metal enhanced fluorescence (MEF),^4^ which permitted new biolabelling approaches based on natural fluorescent molecular sensing within biomembranes in the presence of deposited silver nanoparticles at the right distance.^5^ In this manner, the importance of studying different energy pathways within biostructures *via* quantum experimental approaches was shown as well. Recently it was reported how energy transfer by photosynthetic proteins within bacteria produced modifications from the excited state of excitonic superpositions to the basal state with energy migration, suggesting the quantum role of non-classical energies in natural photosynthetic systems.^6^ If the focus is on nanomaterials for biolabelling applications, one should mention the use of biocompatible hybrid nanomaterials from different synthetic and natural sources as for example silica and gold nanomaterials with optical transparent^7^ and optically active properties respectively in addition to biocompatible properties that permit silica being considered as an inorganic collagen,^8^ and gold nanoparticles applied for laser-assisted therapy for controlled CRISPR delivery *in vivo*.^9^ In addition should be highlighted silica nanomaterials and applications based on nanophotonic luminescent nanoparticles with the incorporation of organic fluorescent dyes^10^ within silica nanocomposites for biomedical imaging,^11^ multiple homogeneous immunoassays based on quantum dots–gold nanorods by FRET nanoplatforms,^12^ gold core–shell silica nanoparticles for biosensing^13^ by MEF, silica waveguides by resonant fluorescent core–shell nanoparticles by MEF,^14^ and drug delivery applications *via* a controlled silica porosity.^15^ So, silica has been widely applied and is still a key nanomaterial for nanophotonic developments. However, new biomaterials are being developed, for example based on controlled nano-aggregated biomolecules with fluorescent properties for bioimaging applications.^16^

In particular for this research study, our interest was in cyanobacteria due to their environmental implications and optical active properties. Cyanobacteria or blue-green algae are the dominant phytoplankton group in eutrophic freshwater bodies worldwide. Moreover, climate change has contributed to increases in cyanobacteria occurrence in surface waters, and the risk of harmful algae blooms.^17^ Therefore, many countries or jurisdictions have implemented specific water quality regulations to protect public health and safety. Drinking water quality guidelines related to cyanobacteria are based on maximum acceptable concentrations of toxins (*e.g.*, microcystin-LR) in treated water (*e.g.*, 1.0 μg L^−1^ proposed by WHO in 1999) or high levels of cyanobacterial cells (*e.g.*, ≥100 000 cells per mL) in water supplies (WHO, 2011).^18^ Despite the time required for identification, confirmation, and enumeration of cyanobacterial cells, direct microscopic enumeration is the simplest and most cost-effective method still used. In natural samples, this method involves some limitations such as the weak contrast of cells against the background, high species diversity, variable morphology of individual cells, and complexity of cell aggregates or units (colonies, entangled filaments *etc.*).

Indirect quantification methods also have been developed to estimate cyanobacterial cell concentrations in water, such as flow cytometry, antibody-mediated immunofluorescence microscopy assays, PCR-fluorescent fragment detection, qPCR molecular probes using sandwich hybridization, and *in situ* fluorescence. However, all of them have some drawbacks, and cost-effective, fast, and reliable cyanobacterial cell identification and enumeration methods are thus much needed.^19^

Therefore, imaging-based enumeration methods appear to be promising for rapid and low-cost water quality monitoring of cyanobacteria, and fluorescent silica nanoparticles could be a means to improve the detection limits and sensitivity of these methods.

Moreover, these types of bacteria were evaluated as optically active biostructures^20^ that interact by non-classical light pathways with luminescent nanoplatforms for potential biotechnological applications^21^ as well.

For these reasons, our interest was focused in the design and synthesis of tunable hybrid nanomaterials based on fluorescent silica nanoparticles with enhanced properties based on FRET for biolabelling applications.

In this manner, for this research communication fluorescent emission properties were tuned by the incorporation of well overlapped spectroscopic properties and optimal quantum yields from molecular donor–acceptor pairs within silica nanoparticles. These nano-emitters were applied for non-covalent cyanobacteria labelling and detection by enhanced fluorescence imaging recorded with laser fluorescence microscopy. Then, evaluation was by in-flow cytometry with nano-biostructure detection and counting with laser excitation and fluorescence detection.

## 2. Experimental

### 2.1 Apparatus

An Olympus confocal laser scanning microscope, FluoView FV1000, was used for fluorescence microscopy imaging and for bright-field confocal microscopy.

Transmission electron microscopy (TEM), JEM-1230, JEOL, with an operating voltage of 200 kV, was used for determination of nanoparticle size.

UV-visible and spectrofluorimetric determinations were carried out with a Varian UV-50 Cary 50 Conc. and a Cary-Eclipse respectively. Lifetime measurements were done with a PicoQuant FluoTime 2000.

The flow cytometer was from BD, model FACSCalibur, with laser excitation at 488.0 nm and 555.0 nm with standard filters at 533/30 and 585/40 for Alexa Fluor 488-A and AF5555-a.

An ultrasonic bath (Branson 2510) was used for the dispersion of the reagents and colloids. Centrifugation was done using an Eppendorf Centrifuge 5804 (range 7500–8000 rpm).

Data analysis was performed with Origin (Scientific Graph system), version 8.

### 2.2 Reagents

Water was obtained using a Millipore apparatus. Other reagents were rhodamine B (RhB) and fluorescein (Fl) (99% purity, Sigma-Aldrich), *tetraethyl orthosilicate* (TEOS) (98%, Sigma-Aldrich), ethanol (Sintorgan, HPLC grade), 3-(aminopropyl)triethoxysilane (APS) (98%, Sigma-Aldrich), *N*-hydroxysuccinimide (NHS) and *N*-(3-dimethylaminopropyl)-*N*′-ethylcarbodiimide hydrochloride (EDC) (98%, Sigma-Aldrich) and sodium cyanide (95%, Sigma-Aldrich).

Ultra-filtrated and deionized water was obtained using a Millipore apparatus.

Micrometer multicolor beads from BD Company were used as control particles for in-flow cytometry.

The wild population of *Microcystis aeruginosa* was concentrated from an atoxic bloom collected in San Roque water reservoir (Cordoba, Argentina).

### 2.3 General procedures

Silica nanoparticles were synthesized based on the classical Störber method.^22,23^ In order to do that, the TEOS concentration was adjusted by variable volume addition of μL aliquots into basic ethanol solution (pH = 9.00) adjusted with concentrated ammonium hydroxide. For the TEOS reaction, variable μL aliquots of TEOS were added, maintaining constant the ratios of reagents as described in the following. The ratio of TEOS/ethanol/H_2_O/NH_4_OH was 150/2300/80/620. For example, for a typical synthesis of varied sizes of silica nanoparticles, 20.0, 40.0 and 80.0 μL were added of a concentrated solution of TEOS = 0.215 ± 0.03 M in 2.5 mL final volume. In this manner were obtained concentrated colloidal dispersions of silica nanoparticles of varied sizes. The average sizes of nanoparticles were 200.0 nm, 240.0 nm, and 380.0 nm, respectively. The errors associated with the determinations of sizes were in general average values of ±5 nm for smaller sizes (200.0–300.0 nm), and ±10 nm for larger sized nanoparticles (350.0–450.0 nm).

**Scheme 1:**
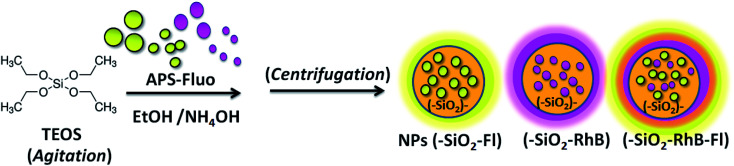
A schematic diagram showing the synthesis of fluorescent silica nanoparticles (NPs–(SiO_2_–Fluo)) *via* the covalent linking of fluorescein (Fl) and rhodamine B (RhB) to obtain mono-coloured NPs–(SiO_2_–Fl) and NPs–(SiO_2_–RhB) and multi-coloured NPs–(SiO_2_–RhB–Fl).

Fluorescent silica nanoparticles were obtained by incorporation into the described silica nanoparticle synthesis fluorophores covalently bonded to modified organosilanes dissolved in ethanol (Scheme 1). The covalent linking of Fl and RhB was done by activation of their carboxylic groups with NHS/EDC and nucleophilic attack from the amine group of APS.^13^ For a typical reaction, 2 mg of RhB or Fl was added in the presence of 10 times higher concentrated APS and NHS/EDC in 2.0 mL total volume of ethanol. Thus, APS–Fl and APS–RhB were obtained.

The silica nanoparticles were centrifuged at 8500 rpm and redispersed in anhydrous ethanol.

The concentrations of fluorophores loaded within the nanoparticles were determined by static fluorescence. These values were obtained from the difference of the known APS-fluorophore concentration added and the concentration of the non-incorporated APS-fluorophores collected from the supernatant of the colloidal dispersion media of the synthesis. So, calibration curves were made for the concentration of APS-fluorophores used for the synthesis of the different fluorescent silica nanoparticles. At the same time the determined values were corroborated by absorption measurements. Moreover, additional controls for dye content within coloured silica nanoparticles were developed by disintegrating the nanostructures in strong (pH = 1) acid media and sonication for a period of 4 h, and then quantifying the respective fluorescent dyes incorporated.

After the synthesis of the fluorescent silica nanoparticles, their emission was controlled to regular levels before use. The control was done by static fluorescence and laser fluorescence microscopy. To do that, the samples were centrifuged and controlled the presence of nanoparticles in the supernatant by dynamic light scattering (DLS). Thus was evaluated the fluorophore leakage from the cleaned supernatant, and the standard emission intensity of nanoparticles from the re-suspended sample. No leakage was recorded for the samples used in the different experiments. And the percentage of variation within different measurements for the same samples was below ±5%. In this manner, were investigated samples with low background emissions determined by laser fluorescence microscopy.

Fluorescence emission spectra were measured with an excitation wavelength equal to the wavelength of maximum absorption of the fluorescent dyes (*λ*_exc_ = 480.0 and 539.0 nm for Fl and RhB respectively). In order to confirm that the maximal emission fluorescence was measured in these conditions, the excitation wavelength was evaluated by measuring 3D fluorescence emission spectra. For emission and excitation fluorescence spectra, the excitation and emission bandwidths were set at 10 nm. The PMT gain was medium. All the measurements were performed at (25.0 ± 0.1) °C, with the temperature of the cell compartment being controlled with a Haake K10 circulator under continuous stirring.

The fluorescence lifetime decay measurements of the fluorescent silica nanoparticles were performed in ethanol.

The cyanobacteria bloom *Microcystis aeruginosa* was concentrated from a sample collected in San Roque water reservoir (Cordoba, Argentina). Cell counts of *Microcystis aeruginosa* were performed with a standard optical microscope using a haemocytometer and then followed by measuring optical density (OD) at 600 nm (OD of 0.1 corresponds to a concentration of 10^8^ cells per mL). The conservation of these samples was performed in diluted (1/10) phosphate-buffered saline (PBS buffer) aqueous solution used for typical DNA hybridization assays.

From this concentrated colloidal dispersion of bacteria, dilutions were done at intermediate and diluted concentration levels (0.2 and 0.05 OD values at 600 nm respectively). Each inoculum was examined with the microscope to confirm its composition and the dominance of *Microcystis aeruginosa* within the sample (>99% cells counted correspond to these cyanobacteria).

**Scheme 2:**
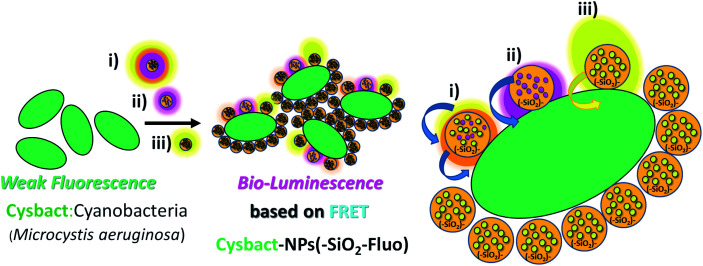
A schematic diagram showing the biolabelling of cyanobacteria (cysbact) with fluorescent mono-coloured SiO_2_–Fl and SiO_2_–RhB NPs and multi-coloured SiO_2_–RhB–Fl NPs, by non-covalent interactions.

For cyanobacteria–nanoparticle interaction, a dispersion of cells was prepared from the bloom sample of *Microcystis aeruginosa*. The bacterial concentrations were determined by measuring OD at 600 nm at intervals of 30 min (OD of 0.1 corresponds to a concentration of 10^8^ cells per mL). In this manner, from a concentrated dispersion of cyanobacteria in aqueous media, variable dilutions were prepared depending on the number of biostructures intended to be determined by the optical microscopy techniques used. Thus, by bright-field confocal microscopy as control, from individual bacteria were obtained micro-aggregates of bacteria. For fluorescent labelling, the dispersions were in contact with variable additions of μL aliquots of concentrated fluorescent silica nanoparticles for a 4 h period of time (Scheme 2). For typical cell labelling, 0.5 mL of concentrated sample in water was added into 2.0 mL of colloidal dispersion (total volume of 2.5 mL). The concentrations of fluorescent nanoparticles as nanolabellers were within the interval of 9 × 10^8^ to 10^10^ NPs per mL depending on the cyanobacterial bloom concentration used.

After that the samples were observed by laser fluorescence microscopy with a minimal volume added (1 drop of 20 μL) on a microscope glass slide (covered after addition with a cover-glass). For this technique was applied laser excitation at 488.0, 543.0 and 555.0 nm. The emission filter bands were placed in the interval of emission wavelengths of 510–625 nm and 575–650 nm for 488.0 nm laser excitation, and within the interval of 575–650 nm for 543.0 nm and 555.5 nm laser excitations.

For the in-flow cytometry analysis, contour plots of side-scattered light (SSC; proportional to cell granularity or internal complexity) *vs.* forward-scattered light (FSC; proportional to cell-surface area or size) were used to characterize distributions of fluorescent event detections. Laser excitations at 488.0 nm and 555.0 nm with emission filters of 530/30 nm and 585/42 nm were used.

The nano-imaging was recorded by variable look-up table image edition (LUT) of brightness and contrast parameters. For bio- and nano-imaging, green, red-green and fire LUT were applied, depending on the degree of detail tracked. For images generated with higher contrast with the background, green and fire LUT were used; while for differentiated intensities recording, red-green LUT was used. For the image edition, the background signal was subtracted that never overcame 10% of the higher intensity collected.

## 3. Results and discussion

### 3.1 Characterization of mono-coloured and multi-coloured fluorescent silica nanoparticles

**Fig. 1:**
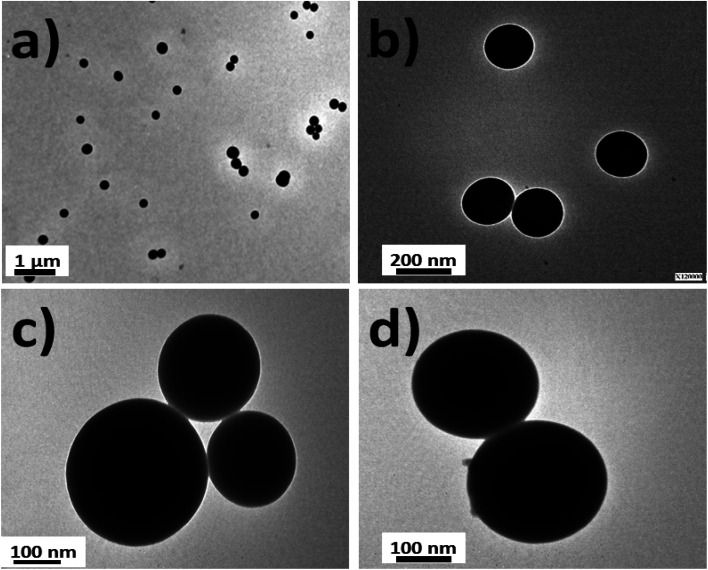
TEM images of silica nanoparticles: (a) the homogeneous distribution of silica nanoparticles; (b) individual silica nanoparticles of 180–200 nm in diameter; (c) silica nanoparticle trimers; and (d) the dimeric nanoparticle distribution.

Silica nanoparticles were obtained by the Störber method with varied diameters depending on the added TEOS concentrations. Sizes of 380.0, 250.0 and 200.0 nm were observed by TEM. From all batches of colloidal dispersions were observed homogeneous silica nanoparticles (Fig. 1a), with well-defined spherical shapes (Fig. 1b) accompanied by small trimer and dimer formations (Fig. 1c and d). The addition of the fluorescent dyes was done by their conjugation with APS for incorporation within the polymerized TEOS organosilane by covalent linking.

**Fig. 2:**
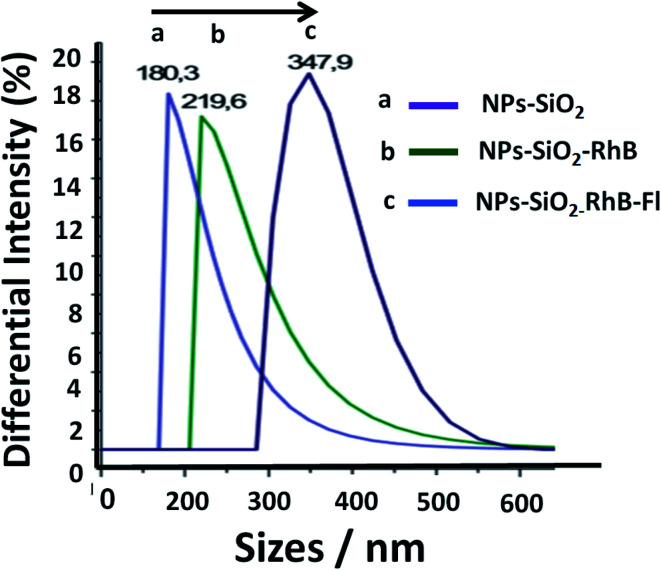
Distribution of sizes in the colloidal dispersion of silica nanoparticles from DLS: (a) non-fluorescent silica nanoparticles (NPs–SiO_2_), (b) mono-coloured silica nanoparticles with rhodamine B (NPs–SiO_2_–RhB) or fluorescein (NPs–SiO_2_–Fl), and (c) multi-coloured fluorescent silica nanoparticles (NPs–SiO_2_–RhB–Fl). The concentrations incorporated within the silica nanoparticles were 0.39 and 0.27 μM for [RhB] and [Fl], respectively.

The fluorescent hybrid nanoarchitectures produced increased in size in the interval of 10 to 40% depending on the added conjugated fluorophore concentrations. For example, silica nanoparticles of 180–200 nm ranges of diameters were incorporated with [RhB] and [Fl] of 0.39 and 0.27 μM respectively, which produced increased sizes of 20% for mono-coloured to 40% for multi-coloured nanoparticles (Fig. 2).

Moreover, the one distribution of well-shaped Gaussians recorded from these nanoparticles showed the good dispersibility in ethanol as well as in aqueous colloidal dispersions. In order to verify the stability of the obtained nanoparticles, different measurements were recorded within a 10 minute period of time. Thus, the intensities and sizes of nanoparticles were stable in the mentioned period of time. For longer periods of time the intensities diminished due to the reduced number of detected nanoparticles. However, it should be highlighted the fast dispersibility of these samples by just shaking them. The zeta-potential measurements were in the interval of −20 to 30 mV. These measurements correlated with typical values from well-dispersible free gold and silver core–silica shell nanoparticles as well.^4,24^

**Fig. 3:**
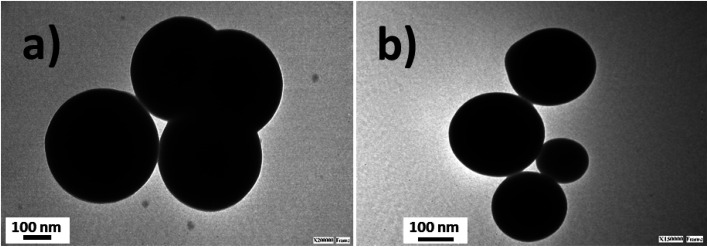
TEM images of fluorescent silica nanoparticles: (a) multi-coloured fluorescent silica nanoparticles (NPs–SiO_2_–RhB–Fl). The range of diameters is 330–350 nm; (b) mono-coloured silica nanoparticles with rhodamine B (NPs–SiO_2_–RhB) or fluorescein (NPs–SiO_2_–Fl). The range of diameters is 230–240 nm.

Moreover, the sizes determined by DLS correlated with determinations by TEM images (Fig. 3). Moreover, it should be mentioned that the addition of the fluorescent dyes did not modify the original spherical shapes of the different hybrid nanoparticles. For NPs–SiO_2_–RhB–Fl, sizes within the 330–350 nm range were recorded; while for NPs–SiO_2_–RhB and NPs–SiO_2_–Fl, average values were within 230–240 nm.

**Fig. 4:**
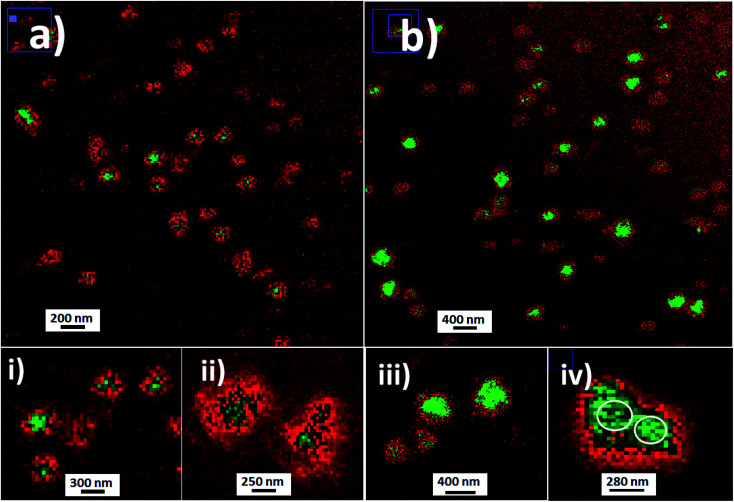
Laser fluorescence microscopy images of fluorescent silica nanoparticles: (a) fluorescein (Fl) mono-coloured silica nanoparticles (SiO_2_–Fl) 180 nm in diameter; (b) modified multi-coloured silica nanoparticles with the incorporation of Fl as a fluorescent energy donor and rhodamine B (RhB) as a resonant energy acceptor. Inset images: (i) and (ii) zoomed images of individual SiO_2_–Fl nanoparticles; (iii) and (iv) zoomed images of individual and dimeric SiO_2_–RhB–Fl nanoparticles. Image edition with red-green color LUT.

Then by laser fluorescence microscopy, the nanoparticles obtained were evaluated. In this manner were recorded strong variable emissions from fluorescent nanoparticles with variable dimensions depending on the fluorophores incorporated and the emission pathways involved. From fluorescent silica nanoparticles modified with Fl (SiO_2_–Fl) were recorded strong emission intensities from reduced sizes close to individual nanoparticle dimensions determined by TEM and DLS. Moreover NPs–(SiO_2_–Fl) nanoparticles showed good dispersibility and homogeneous nanostructures were detected (Fig. 4a); however more enhanced fluorescent nanoparticle detections were recorded from multi-coloured fluorescent silica nanoparticles (Fig. 4b).

About the stability and well-dispersible characteristics of the nanoparticles obtained within colloidal dispersions, it should be mentioned that the re-dispersion of decanted nanoparticles was done easily by simply manual shaking. So, for example, a fast re-dispersion of decanted nanoparticles in glass vials with a clear transparent and limpid solution was modified to bright yellow-orange colloidal dispersions for NPs–(SiO_2_–RhB–Fl), yellow for NPs–(SiO_2_–Fl), and purple for NPs–(SiO_2_–RhB) nanoparticles. Moreover, this phenomenon was observed as well by a change of opalescence from non-coloured silica nanoparticles.

The NPs–(SiO_2_–Fl) emission intensities showed bright and clear dots at 225.0 nm (inset (i) in Fig. 4). These nanoparticles produced a strong green fluorescent core surrounded with gradually diminished red fluorescence intensities (inset (ii) in Fig. 4) from dual-coloured red-green LUT image edition. However their intensities were lower than those of multi-coloured silica nanoparticles with the incorporation of RhB and Fl (SiO_2_–RhB–Fl) (inset (iii) in Fig. 4). The multi-coloured NPs–(SiO_2_–RhB–Fl) nanoparticles showed increased emissions of at least 35% accompanied by the generation of more enhanced fluorescent surfaces (inset (iv) of Fig. 4) in comparison to mono-coloured silica nanoparticles. The enhanced emitter surface core green highlight generated from multi-coloured NPs–(SiO_2_–RhB–Fl) nanoparticles was explained by an improved fluorescence energy routing through the 3D silica nanostructure after interaction with the laser beam. In this manner were recorded higher emission intensities from more highly luminescent surfaces that generated bigger sizes of nanoparticles recorded by laser fluorescence microscopy (Fig. 4) than by TEM (Fig. 3).

Moreover, it should be mentioned that by single fluorescence nanoparticle analysis, the mono-coloured and multi-coloured fluorescent nanoparticles showed homogeneous distributions of emission intensities as it was previously described. For this reason, at this point it should be clarified that the observed emission differences from the nanoparticles within colloidal dispersions (Fig. 4a and b) were attributed to nanoparticles randomly detected in Brownian motion in different planes and deep within a confined μL-volume drop added on the glass microscope slide.

Moreover, it should be highlighted that the distributions of dimeric SiO_2_ nanoparticles based on non-covalent interactions previously mentioned (insets (iii) and (i) of Fig. 4) were explained by an optimal ratio of sizes and interaction strength. Due to the chemistry involved in the developed silica nanoparticles, the contributing forces were polar non-covalent interactions from the hydroxyl groups of silanol accompanied as well by attractive van der Waals interactions. These non-covalent interactions generated from nano-surfaces could be explained by Hamaker constants.^25,26^ The Hamaker constant considers the ratio of non-covalent interaction force and the available nano-surface in contact.^27^ In this manner, for example, were reported forces between dimers of larger sized polystyrene beads in the range between 0.3 and 50 pN in the presence of controlled ionic strength.^28^ Thus, the higher frequency of dimeric forms of the silica nanoparticles obtained by us was explained as due to an optimal ratio of available surface and forces for intermediate particle sizes of 200–300 nm, and not just obtained by Brownian motion and encountering. This fact prompted our interest to study potential applications of dimeric forms by chemical modification of the nano-surfaces with short molecular spacers^29–31^ for non-classical light generation^32^ and nano-resolution.^33^

**Fig. 5:**
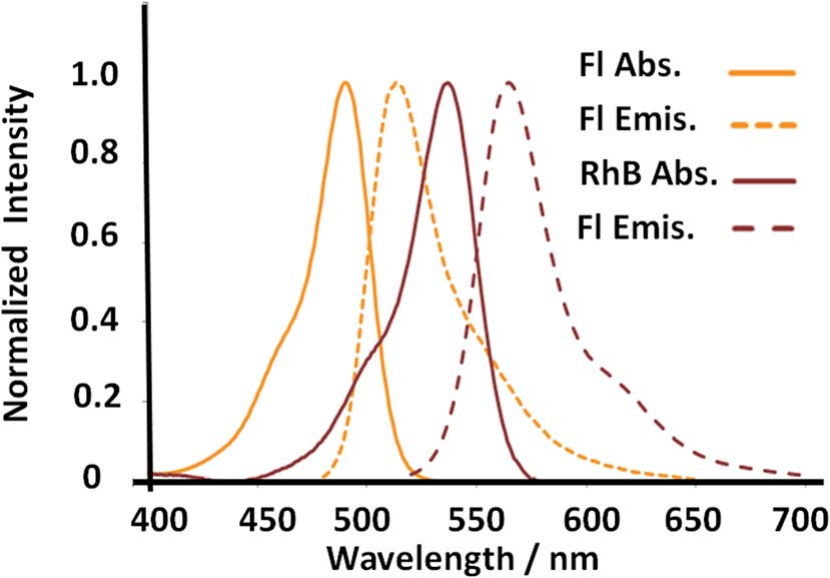
Absorption and fluorescence emission properties of the donor and acceptor dyes incorporated within multicolor gold core–shell nanoparticles.

In addition, the enhanced surfaces accompanied by higher emission properties from SiO_2_–RhB–Fl nanoparticles were explained by FRET.^34^ Both fluorophores showed well overlapped spectroscopic properties from the emission of the energy donor (Fl) to the absorption of the energy acceptor (RhB) (Fig. 5). In addition, for the fluorescent energy donor^35^ were reported three times higher quantum yields than for the energy acceptor.^36^ This enhancement occurred only when the samples were excited at 488.0 nm that corresponded to the maximal absorption of Fl as fluorescent energy donor. While for 543.0 nm and 555.0 nm laser excitations, for only the energy acceptor RhB stimulation, diminished emissions were recorded in comparison to 488.0 nm laser excitation. Moreover, controls of mono-coloured silica nanoparticles showed drastic reductions of their emissions in comparison to multi-coloured nanoparticles. These phenomena *via* FRET pathways were previously studied by us.^37^

The observations achieved by laser fluorescence microscopy were recorded with emission filters in the interval of emission wavelengths of 510–625 nm for 488.0 nm laser excitation to record the complete emission band of both fluorophores considering all the fluorescence emission phenomena as individual fluorophore emission and coupled phenomena *via* FRET. Similar observations were recorded when the interval of the emission filters was changed to longer emission wavelengths, as 575–650 nm (with 488.0 nm laser excitation), to diminish the contribution of the Fl fluorescent reporter and increase the contribution of the emission from the FRET pathway. However, for 543.0 nm and 555.5 nm laser excitation of only the RhB fluorescent reporter with longer interval of emission window, at 575–650 nm, their emission intensities were drastically diminished in comparison to 488.0 nm laser excitation.

**Fig. 6:**
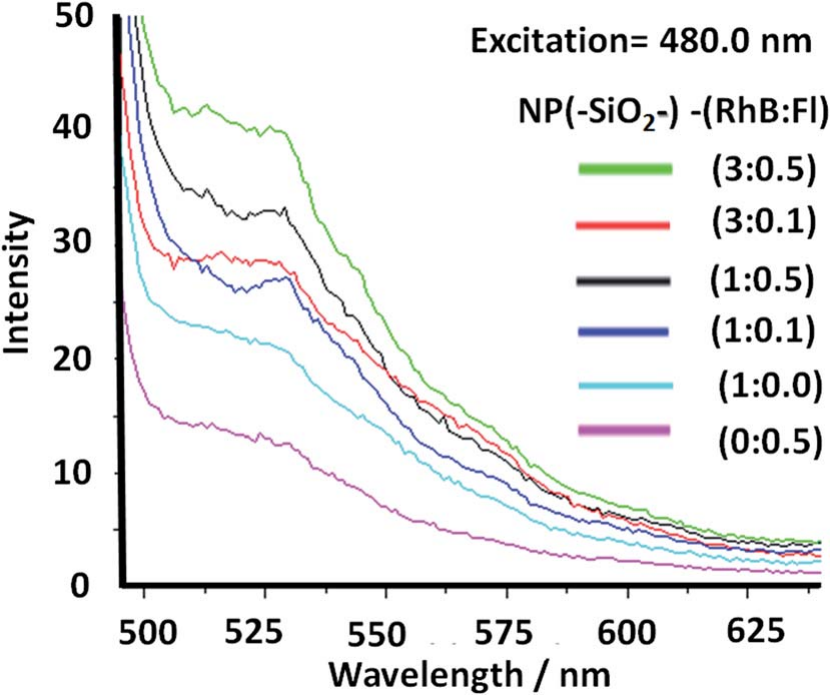
Static fluorescence emission of multi-coloured silica nanoparticles with different ratios of rhodamine B (RhB) and fluorescein (Fl) concentrations (NP(SiO_2_–)–RhB : Fl). The ratio 1 : 1 corresponds to 1.0 μM. The excitation wavelength, applied at 480.0 nm, corresponds to the optimal excitation of Fl as a fluorescent energy donor reporter.

In order to tune the optimal emissions considering the well-known quenching by intermolecular energy homo-transfer for Fl^38^ and RhB,^39^ the concentration of both fluorescent laser dyes was varied. Then in optimal concentration conditions for maximal emissions, and neglecting reduction of emission by quenching in confined volumes, the ratio of concentrations between RhB and Fl (ratio of RhB : Fl) was varied. In this manner with optimal excitation of Fl as fluorescent energy donor, variable fluorescence emission was recorded depending on RhB : Fl ratio (Fig. 6) within the interval of emission wavelengths of Fl and RhB. From a RhB : Fl ratio = 1 : 0 with mono-coloured SiO_2_–RhB NPs to RhB : Fl ratio = 1 : 0.1 and 1 : 05 with multi-coloured SiO_2_–RhB–Fl NPs, higher emissions were recorded with increasing concentrations of Fl than for mono-coloured SiO_2_ NP controls. As reference value the ratio 1 : 1 corresponded to the incorporation of a total dye concentration of 1.00 ± 0.04 μM. In this manner, multi-coloured silica nanoparticles with a ratio of RhB : Fl = 1 were incorporated with concentrations of each component in the interval of 0.4–0.6 μM.

From the fluorescence measurements the ratio of emissions was evaluated within different intervals of wavelengths attributed to RhB and Fl at the different ratio of concentrations of both fluorophores incorporated.

In this manner, the ratios of intensities between the emissions of Fl and RhB within the SiO_2_–RhB–Fl NPs were Fl/Fl_ref._ = 1.7 and 2.3 for RhB : Fl ratio = 1 : 0.1 and 1 : 05 respectively. While the ratios RhB/RhB_ref._ were 1.1 and 1.5 (Table 1). As could be observed from these results, both emissions bands had higher emission than for mono-coloured SiO_2_ NPs.

**Table 1:** Fluorescence emission band ratios of multi-coloured and mono-coloured silica nanoparticles

SiO_2_ NPs^a^	Ratio [RhB]/[Fl]^b^	(Int_[em]_/Int_[em]_ ref.)^c^		
		(Fl/Fl_ref._)	(RhB/RhB_ref._)	(RhB/Fl)
NP(SiO_2_–)–RhB : Fl^d^	3.0 : 0.5	1.9	1.2	0.9
	3.0 : 0.1	**3.0**	**1.7**	0.7
	1.0 : 0.5	**2.3**	**1.5**	1.0
	1.0 : 0.1	1.7	1.1	1.0
NP(SiO_2_–)–RhB–(—)^d^	1 : 0	—	**1.0**	—
NP(SiO_2_–)–(—) : Fl^d^	0 : 0.5	**1.0**	—	—

a Silica nanoparticles (SiO_2_ NPs) with the incorporation of fluorescein (Fl) and rhodamine B (RhB) fluorophores.

b Different ratios of [RhB] and [Fl] concentrations incorporated within NP(SiO_2_–)–RhB : Fl. The ratio 1 : 1 corresponds to 1.0 μM.

c Ratio of intensities of emission bands of Fl at 520.0 nm and RhB at 560.0 nm.

d NP(SiO_2_–)RhB : Fl corresponds to multi-coloured NPs, and NP(SiO_2_–)RhB and NP(SiO_2_–)Fl correspond to mono-coloured NPs. The excitation wavelength applied was 480.0 nm, which corresponds to the optimal excitation of Fl as a fluorescent energy donor reporter.

Moreover, for RhB : Fl ratios of 3 : 0.1 and 3 : 0.5, ratios of intensities between the emissions of Fl in the presence and absence of RhB (Fl/Fl_ref_) of 3.0 and 1.9 respectively were observed (Table 1). So, the tendency showed a diminution and opposite direction in comparison to lower concentration of RhB as energy acceptor. This trend was produced by the quenching effect from dimeric species of both fluorophores at higher concentrations of RhB and Fl.

In this way should be highlighted the ratio of intensities between RhB and Fl (RhB/Fl) emission bands increasing the RhB concentrations from RhB : Fl = 1.0 : 0.5 to 3.0 : 0.5, and 1.0 : 0.1 to 3.0 : 0.1 that generated a reduction of their emissions (Table 1). So, considering only increasing the concentration of RhB as energy acceptor, the emissions were reduced caused by homo-transfer and quenching. It is known that for higher concentrations of these dyes, homo-transfer was produced due to the close intermolecular proximity accompanied by the formation of quenched dimeric species.^40^ This phenomenon was shown by other similar derivatives such rhodamine 6G adsorbed on titanium oxide nanoparticles depending on the added concentration.^41^

However, tuning concentrations to obtain the right ratio of RhB : Fl was required to obtain FRET pairs. The RhB/Fl emission bands were controlled and maximized within confined silica nanoparticles avoiding the quenching contribution.

**Fig. 7:**
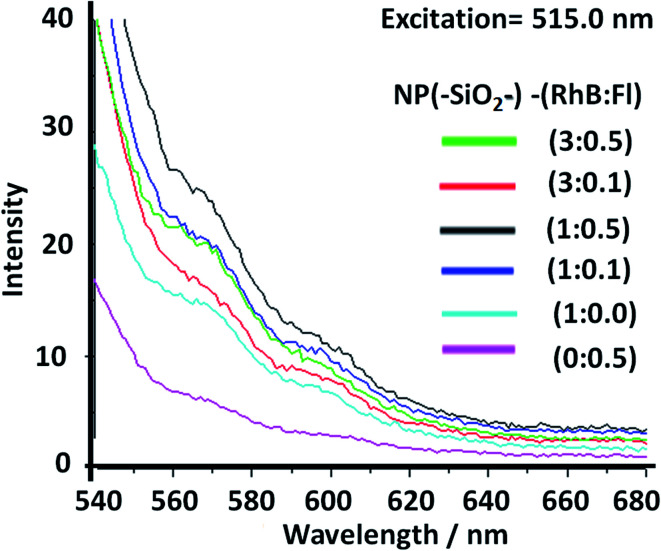
Static fluorescence emission from multi-coloured silica nanoparticles with different ratios of rhodamine B (RhB) and fluorescein (Fl) concentrations. The ratio 1 : 1 corresponds to 1.0 μM. The excitation wavelength applied was 515.0 nm, which corresponds to improved RhB as a fluorescent energy acceptor and partially diminished excitation of Fl as a fluorescent energy acceptor.

Then, with excitation at 515.0 nm, for optimal excitation of RhB as fluorescent energy acceptor and partially Fl as fluorescent energy donor, noted were diminished emissions accompanied by small intensity increase with addition of higher Fl concentrations. Moreover for higher RhB concentrations a quenching effect was observed. In this manner it was shown how by controlling the excitation wavelength it was possible to activate or deactivate different fluorescent emission pathways within a confined volume at the nanoscale with incorporation of different emitters (Fig. 7).

In this manner these nanoparticles showed excellent properties for nano-tracking and biolabelling applications based on their tuneable emission properties. Moreover it should be highlighted that these silica nanoplatforms showed potential chemical surface modifications for bioconjugation by covalent and non-covalent interactions based on their polar surfaces given by hydroxyls of silanol groups. At this point, it should be mentioned for example that already reported were interactions of aminated and thiosulfonated modified silica nanoparticles with *Escherichia coli*,^42^ permitting homogeneous deposition of the nanoparticles over these biostructures. In this study was shown the implication of non-covalent interactions such as polar interactions, van der Waals forces, and strong hydrogen bonding between the biomolecules placed on the bacterial membrane and the modified hydroxyl groups and free silanols.

For these reasons their applications were evaluated as nano-labellers for cyanobacteria labelling.

### 3.2 Bioimaging based on fluorescent cyanobacteria nano-labelling

In order to apply these fluorescent silica nanoparticles for cyanobacteria labelling, variable aliquots of bacteria were added within concentrated conditions of the different optimized fluorescent nano-labellers. To do that were chosen smaller nanoparticle sizes of 180–200 nm due to their improved resolution at the nanoscale accompanied by strong emission intensities and stronger non-covalent interactions between nanoparticles based on their van der Waals interactions predicted from Hamaker constants.^43^ In addition, exo-cellular polysaccharides produced from cyanobacteria^44^ generated, in the absence of nano-labellers, strong inter-cyanobacteria interactions. While in the presence of the modified silica nanoparticles additional strong hydrogen bridges could be involved in their interactions and targeted depositions over the biostructures. So, based on strong van der Waals, polar and non-covalent interactions, and hydrogen bridges between cyanobacteria and silica nano-labellers, their interactions were evaluated by different microscopy methodologies. In this manner smaller fluorescent nano-labeller sizes showed better bio- and nano-surface ratio. In this manner could be deposited a higher number of nanoparticles per biostructure. However, it should be mentioned that larger sizes interacted as well with the cyanobacterial biostructures.

**Fig. 8:**
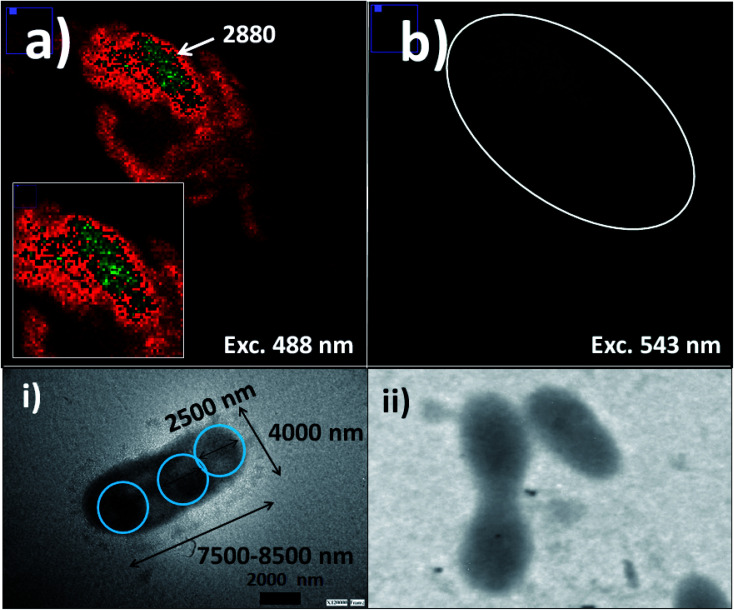
Cyanobacteria labelling with fluorescent silica nanoparticles: (a) laser fluorescence microscopy imaging of labelled cyanobacteria with SiO_2_–Fl nanoparticles with excitation at 488.0 nm; (b) laser fluorescence microscopy imaging of labelled cyanobacteria with SiO_2_–RhB nanoparticles with excitation at 555.0 nm. Inset images: (i) and (ii) TEM images of labelled cyanobacteria with SiO_2_ and non-labelled cyanobacteria, respectively. For the laser fluorescence microscopy image edition, a red-green and green color LUT was applied for optimal results depending on the emission intensities from the nano-biostructure.

Thus was observed by laser fluorescence microscopy the generation of bioimaging from small cyanobacterial aggregates formed from tetramers to higher nano-bio-aggregates. Cyanobacterial labelling with NPs–(SiO_2_–Fl) showed enhanced emission from labelled bio-surfaces with optimal laser excitation at 488.0 nm. LUT edition image with dual red-green color permitted obtaining the variations of the emission intensities from the nano-biostructures (Fig. 8a). The stronger green intensities corresponded to homogeneous NPs–(SiO_2_–Fl) nano-labeller depositions; while the multi-coloured silica nanoparticles produced quenched emissions as well as in the presence of labelled cyanobacteria with NPs–(SiO_2_–RhB) (Fig. 8b) at both laser excitations applied. By optimal excitation of RhB at 543.0 and 555.0 nm, homogeneous low emission was recorded from the labelled biosurfaces (Fig. 8b) as was observed for NPs–(SiO_2_–RhB) biolabelling. But, from non-labelled cyanobacteria drastically diminished bioimaging was recorded due to their intrinsic low fluorescence emissions.^45^

The deposition of silica nanolabellers was corroborated by TEM (inset image (i) of Fig. 8) by increased contrast from labelled bacteria in comparison to non-labelled cyanobacteria (inset image (ii) of Fig. 8).

**Fig. 9:**
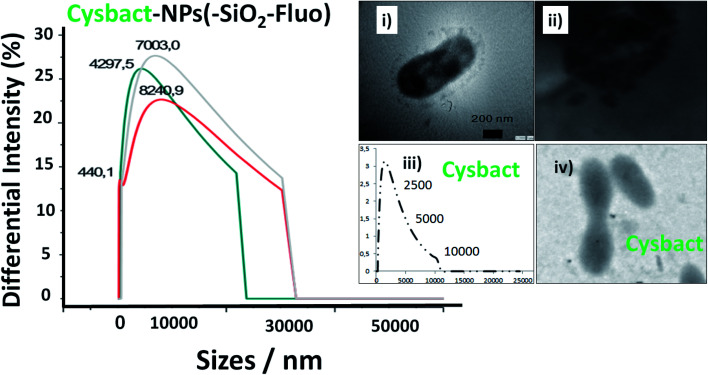
Distribution of sizes by DLS of labelled cyanobacteria with fluorescent silica nanoparticles within a 10 minute interval of time. Blue, grey, and red lines corresponded to 1, 5, and 9 minutes, respectively. Inset images: (i) a TEM image of an individual labelled cyanobacterium; (ii) a TEM image of aggregated labelled cyanobacteria; (iii) the distribution of sizes of non-labelled cyanobacteria (cysbact) by DLS; and (iv) a TEM image of non-labelled cyanobacteria (cysbact).

Moreover, DLS measurements showed higher distribution of labelled bacterial sizes than non-labelled cyanobacteria. Smaller average sizes within 5000.0–7500.0 nm range and larger aggregates as well were recorded by DLS (Fig. 9). The sizes were verified by TEM that corresponded to labelled single, dimeric and trimeric cyanobacteria (inset (i) of Fig. 9), and larger aggregates (inset (ii) of Fig. 9). The labelled biostructure sizes were larger than the non-labelled ones (inset (iii) of Fig. 9) measured by DLS. The labelled cyanobacteria showed higher contrasted images than non-labelled biostructures (inset (iv) of Fig. 9). This fact was explained by the presence of coloured silica nanoparticles with higher electron density from the highly conjugated chromophores incorporated within the silica nanoparticles.

**Fig. 10:**
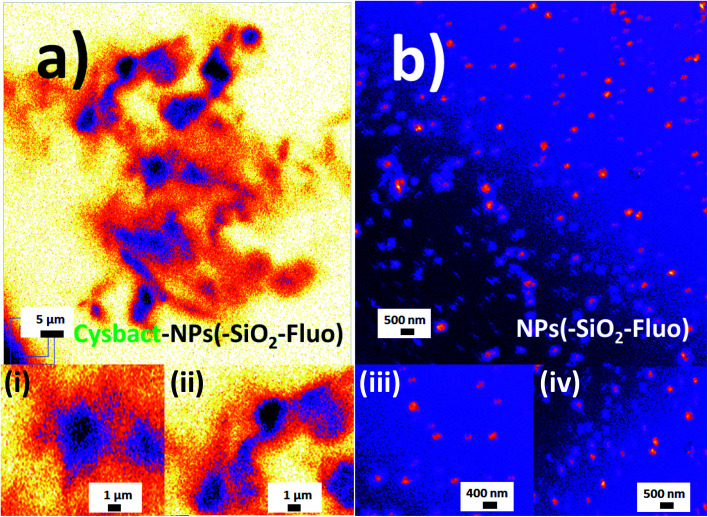
Laser fluorescence microscopy: (a) aggregated labelled cyanobacteria with mono-coloured SiO_2_–Fl nanoparticles (cysbact–NPs(–SiO_2_–)Fluo); (b) a colloidal dispersion of mono-coloured SiO_2_–Fl nanoparticles. Inset images: (i) and (ii) zoomed images of dimeric and trimeric forms of labelled cyanobacteria, respectively; (iii) and (iv) zoomed images of mono-coloured SiO_2_–Fl nanoparticles. For the laser fluorescence microscopy image edition the fire LUT was applied.

In addition, it should be added that the sizes measured by DLS of the nano-biostructure aggregates with NPs–(SiO_2_–Fl) corresponded to those observed by laser fluorescence microscopy (Fig. 10a). From these hot-spots only were generated stronger emissions. In optimized conditions, non-aggregated free nano-labellers were observed. While, in the absence of the cyanobacteria were observed single free NPs–(SiO_2_–Fl) nano-labellers, and from dimeric to tetrameric species as well (Fig. 10b), but not observed in any case were similar shapes, aggregates and sizes as was observed for the nano-biostructures (Fig. 10b).

**Fig. 11:**
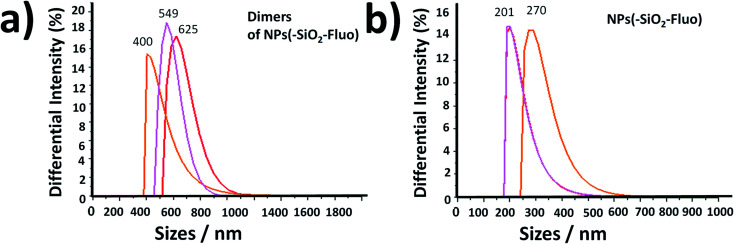
The distribution of smaller sizes in a colloidal dispersion by DLS of (a) dimeric nanoarchitectures of fluorescent silica NPs; and (b) individual fluorescent silica NPs. Size distribution measurements were carried out within 10 minutes. The pink, orange, and red lines correspond to 1, 5, and 9 minutes, respectively.

The free nanolabellers and small nanoaggregates observed were confirmed in colloidal dispersion in the absence of cyanobacteria by DLS measurements. Dimeric and trimeric species of NPs–(SiO_2_–Fl) nanoparticles were determined (Fig. 11a), as well as single nanoparticles (Fig. 11b). These colloidal dispersions were stable within 10 minutes. After this period of time, they showed diminished intensities from decantation in colloidal dispersion. Therefore, this effect diminished the detection of the dispersed nanoparticles in the colloidal dispersion. However, by simple manual shaking they were redispersed. So, the colloidal dispersions were well stable and dispersible.

In this manner was confirmed the use of the fluorescent silica nanoparticles as nano-labellers by non-covalent interactions for cyanobacterial bioimaging.

The strong non-covalent interactions were explained by hydrogen bonding that showed higher strengths than other non-covalent interactions^46,47^ with a high dependence of the electronic donor–acceptor lengths from the functional groups or atoms involved.^48^ This could explain some observations made by us with different real water samples with variable composition and possible interferences. However further experiments should be done in order to study the effect of possible interferences from real matrixes, as well as the possible incorporation of the nanolabellers within the biostructure.

From the literature the fluorescence technique showed high sensitivity for biolabelling, with an example application being the use of mannose-fluorescent functionalized polymer for *Escherichia coli* labelling and detection.^49^ Moreover, modified silica nanoparticles being aminated and thiosulfonated were used for non-covalent depositions on *Escherichia coli*^42^ as previously discussed. Moreover, it should be highlighted the recent development by us of ultraluminescent gold core–shell silica nanoparticles based on MEF for individual *Escherichia coli* detection.^13,50^ In addition, cyanobacterial non-specific labelling with quantum dots^51^ on filamentous structures of cyanobacteria was reported as well. In this way, it should be mentioned that cyanobacteria could generate biofilm formation by their exocellular polysaccharide production and higher aggregates as well.^52^ Thus, in the context of antifouling nanoparticle applications, non-covalent deposition of silanized magnetic nanoparticles has been reported^53^ and PEG–silver nanoparticles^54^ as well as other types of nanomaterials.^55^

So, to the best of our knowledge, the utilisation of silica nanoparticles for non-covalent luminescent biolabelling applications of cyanobacteria has not been reported yet; however non-covalent depositions of different nanoparticles and nanomaterials were already reported.

### 3.3 Static and time-resolved fluorescence characterization by mono- and multi-coloured nano-silica cyanobacteria labelling

Based on the biolabelling methodology described and due to variable emission properties observed for the different mono-coloured and multi-coloured fluorescent silica nanoparticles, their static and time-resolved emissions were evaluated quantitatively.

**Fig. 12:**
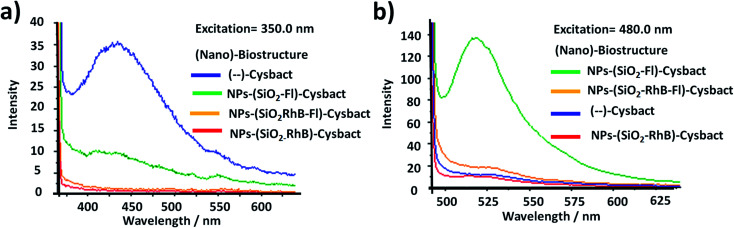
Fluorescence emission of non-labelled and labelled cyanobacteria with fluorescent SiO_2_ nanoparticles (NPs–SiO_2_–Fluo) at (a) an excitation wavelength of 350.0 nm, which corresponds to the maximal absorption wavelength of cyanobacteria; and (b) at an excitation wavelength of 480.0 nm, which corresponds to the maximal absorption wavelength of fluorescein (Fl) as a fluorescent energy donor reporter.

First, it should be mentioned that variable absorption and low-intensity emission were reported for different types of cyanobacteria.^56^ In particular the wild population of *Microcystis aeruginosa* studied showed a higher absorption band in the UV region around 350.0 nm. In concentrated conditions static fluorescence measurements showed 4 times higher emission intensities than cyanobacteria labelled with NPs–SiO_2_–Fl nano-labellers and even higher than with NPs–(SiO_2_)–RhB and multi-coloured NPs–(SiO_2_–RhB–Fl) nanoparticles (Fig. 12a). This fact was clearly explained by the optimal excitation of fluorescent photosystem and negligible absorption from the fluorescent reporters incorporated within the different silica nanoparticles. This fact showed the important role of the fluorophores and control of the light recorded from the non-labelled biostructure and labelled biostructure by the excitation wavelength applied. But, by the optimal excitation of Fl as a fluorescent energy donor reporter at 480.0 nm, up to ten times higher emission intensities were recorded with the application of NPs–(SiO_2_–Fl) nanoparticles than non-labelled cyanobacteria. However, the attendant enhanced fluorescence emission from multi-coloured NPs–(SiO_2_–RhB–Fl) nano-labellers observed from free nanoparticles was not observed. Instead were recorded diminished emissions with only 25% of the increase in comparison to non-labelled cyanobacteria. While with NPs–(SiO_2_–RhB) nano-labellers was observed 10% reduction in comparison to non-labelled cyanobacteria (Fig. 12b). The main fact to explain this was the diminution of the nano-biostructures with NPs–SiO_2_–(RhB–Fl) nano-labellers in comparison to NPs–(SiO_2_–Fl) in the absence of the fluorescent energy acceptor RhB.

In order to complete this study for a well understanding of the emission pathways, fluorescence lifetime decays were measured of the free nano-labellers and labelled cyanobacteria.

For multi-coloured and mono-coloured nanoparticles were recorded bi-exponential fluorescent lifetime decays (*τ*_1_ and *τ*_2_). For mono-coloured silica nanoparticles were recorded a shorter component related to scattering (*τ*_1_) and longer decay (*τ*_2_) related to the confined fluorophores within the silica nanoarchitecture (Table 1). These values correlated with reported literature for free Fl^35^ and RhB^36^ fluorophores slightly modified by scattering. While for multi-coloured silica nanoparticles with optimal excitation of Fl as fluorescent energy donor reporter, a *τ*_2_ shortening of 15% was obtained. This fluorescence lifetime decay shortening was accompanied by 25% and 35–40% emission intensity increases recorded by static fluorescence and laser fluorescence microscopy respectively. This enhancement in the presence of both emitters accompanied by diminished fluorescence lifetime decays supported the improved emission pathway based on FRET already observed and discussed in connection with laser fluorescence microscopy.

**Fig. 13:**
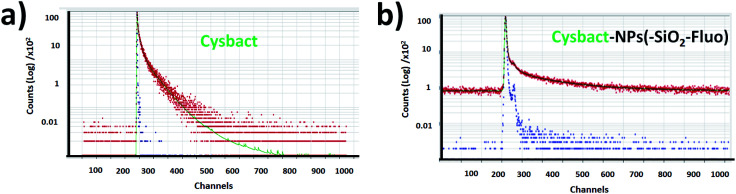
Fluorescence lifetime decays within colloidal dispersions of (a) labelled cyanobacteria with mono-coloured silica nanoparticles by the incorporation of fluorescein (cysbact–NPs(SiO_2_–Fl)) and (b) cyanobacteria (cysbact).

Then from labelled cyanobacteria were recorded multi-exponential decays related to the emission from the different components of the nano-biostructures formed by various chromophores and emitters from the nano-labellers and photosynthetic systems of cyanobacteria. For non-labelled cyanobacteria was recorded tetra-exponential decay fitting (Fig. 13a); *τ*_1_ and *τ*_2_ of 1.53 and 6.120 ns correlated with shorter and longer fluorescent lifetime decays, while *τ*_3_ and *τ*_4_ of 30.62 and 61.24 ns were related to modified emission from more aggregated bacteria (Table 2). As was already reported for these types of cyanobacteria, variable fluorescent lifetime decays were collected depending on their chromophore compositions.^57^ However faster decays related to 0.75 to 3.0 ns interval values were reported for different molecular composition of photosynthetic systems, as well as, depending on their state of aggregation, longer components being recorded.^58^ So, the tetra-exponential fitting showed clearly reduced fluorescence lifetime decays (Fig. 13b) in comparison to non-labelled cyanobacteria (Fig. 13a). Thus, for cyanobacteria labelled with NPs–(SiO_2_–Fl) nanoparticles (SiO_2_–Fl–cysbact) were recorded diminished shorter and longer fluorescent lifetime decays. Values of *τ*_1_, *τ*_2_, and *τ*_3_ of 0.02, 0.120, and 0.99 ns respectively were determined, and *τ*_4_ of 6.10 ns (Table 2). Noted are the reduced values of *τ*_2_ and *τ*_3_ for NPs–(SiO_2_–Fl)–cysbact in comparison to *τ*_1_ and *τ*_2_ for non-labelled cyanobacteria. In addition it should be mentioned that these reductions of lifetime decays were accompanied by 35–40% higher emission intensities from nano-biosurfaces than from free NPs–(SiO_2_–Fl) nano-labellers, with up to 10 times higher emissions than non-labelled cyanobacteria. However, from labelled multi-coloured cyanobacteria with NPs–(SiO_2_–RhB–Fl) nano-labellers, drastic reduction of emission intensities was recorded accompanied by fluorescence lifetime decay shortening. The values of *τ*_1_, *τ*_2_, and *τ*_3_ were even lower than with NPs–(SiO_2_–Fl) nano-labelling (Table 2); however the transferred energy was conducted within a radiative pathway with a lower quantum yield that generated the reduction in intensity.

**Table 2:** Fluorescence lifetime decay measurements of nano-biostructures obtained based on cyanobacteria labelled with mono-coloured SiO_2_–Fl and multi-coloured SiO_2_–RhB–Fl NPs

Nano-biostructure^a^	*τ*_1_ (ns)	*τ*_2_ (ns)	*τ*_3_ (ns)	*τ*_4_ (ns)
(RhB–Fl)^b^	0.04 ± 0.02	**3.65** ± 0.02	(—)	(—)
(Fl)^c^	0.91 ± 0.01	**3.97** ± 0.02	(—)	(—)
(RhB–Fl)–cysbact^d^	0.002 ± 0.001	0.008 ± 0.002	**0.300** ± 0.001	**5.900** ± 0.002
(Fl)–cysbact^e^	0.02 ± 0.01	0.120 ± 0.001	**0.990** ± 0.003	**6.100** ± 0.002
(—)–Cysbact^f^	**1.530** ± 0.003	**6.120** ± 0.002	30.620 ± 0.002	61.240 ± 0.004

a Cyanobacteria of *Microcystis aeruginosa* (cysbact) were evaluated for nano-biolabelling with fluorescent SiO_2_ NPs.

b Free SiO_2_–RhB–Fl NPs with Fl as the fluorescent energy donor and RhB as the resonant energy acceptor with a maximal absorption wavelength of 470.0 nm in the absence of cyanobacteria.

c Free SiO_2_–Fl NPs with Fl as the fluorescent reporter with a maximal absorption wavelength at 480.0 nm.

d Labelled cyanobacteria with NPs–(SiO_2_–RhB–Fl) ((RhB–Fl)–cysbact).

e Labelled cyanobacteria with NPs–(SiO_2_–Fl) ((Fl)–cysbact).

f Non-labelled cyanobacteria ((—)–cysbact).

This fact was correlated with fluorescence emission bands recorded from time-gated fluorescence in intact blue-green and red algae from B-phycoerythrin as intermediate chromophore in their complex photosynthetic systems that showed two emission bands centered at 530.0 and 645.0 nm selectively excited at 540.0 nm.^59^ From this it was observed that up to 4 times higher emissions could be obtained from the emission band centered at 530.0 nm than at 645.0 nm. The mentioned differences showed the relative quantum yields between the chromophores involved in the emissions from non-labelled cyanobacteria that in the presence of the mono-coloured and multi-coloured nano-labellers coupled different bacterial chromophores *via* a bio-FRET pathway. In the presence of mono-coloured NPs–(SiO_2_–Fl) nano-labellers clear enhancements were found explained by the coupling based on the well-overlapping shorter emission band with the cyanobacterial chromophore with higher quantum yield centered at 530.0 nm. While in the presence of the mono-coloured NPs–(SiO_2_–RhB) and multi-coloured NPs–(SiO_2_–RhB–Fl) nano-labellers, the diminished emission was due to the coupling with the lower quantum yield chromophore with the longer wavelength band centered at 645.0 nm. In this manner in the presence of labelled cyanobacteria with RhB emitter was recorded a diminished emission pathway conducted *via* bio-FRET, which in the presence of RhB–Fl pair was slightly enhanced but it was not proportional to the enhancements recorded for free NPs–(SiO_2_–RhB–Fl) nano-labeller.

To the best of our knowledge, there has been no previous report of an enhanced bio-structure like this one by controlling targeted emissions through different quantum yielding natural photo-receptors. However, this is a high-impact research field within biophotonics such as already recently reported for living lasers^60^ based on the incorporation of green fluorescent proteins in cells. Thus the importance was shown of the design of nano-engineered materials as optical gain media^61^ for bio-applications. In a similar manner, it could be mentioned the synthesis and fabrication of new materials for the design of miniaturized devices and instrumentation for targeted light delivery^62^ for sensing and bioimaging^63^ within confined biostructures and tissues.

In summary, the reported luminescent nanoplatforms showed variable and controlled emissions depending on the fluorescent emitter reporters incorporated within the silica nanoparticles. In the presence of the RhB–Fl FRET pairs, enhanced emissions were observed in comparison to mono-coloured nanoparticles. However these enhancements were coupled *via* a bio-FRET pathway with 4–5 times lower cyanobacteria chromophore quantum yield that produced clear diminished emission from multi-coloured and mono-coloured silica nanoparticles in the presence of RhB as fluorescence energy acceptor and emitter (Scheme 2(i) and (ii)). And with mono-coloured NPs–(SiO_2_–Fl) nano-labelling was coupled the higher bacterial chromophore quantum yield that produced enhanced bio-FRET emissions (Scheme 2(iii)).

### 3.4 Cyanobacteria detection by in-flow cytometry and laser fluorescence microscopy

**Fig. 14:**
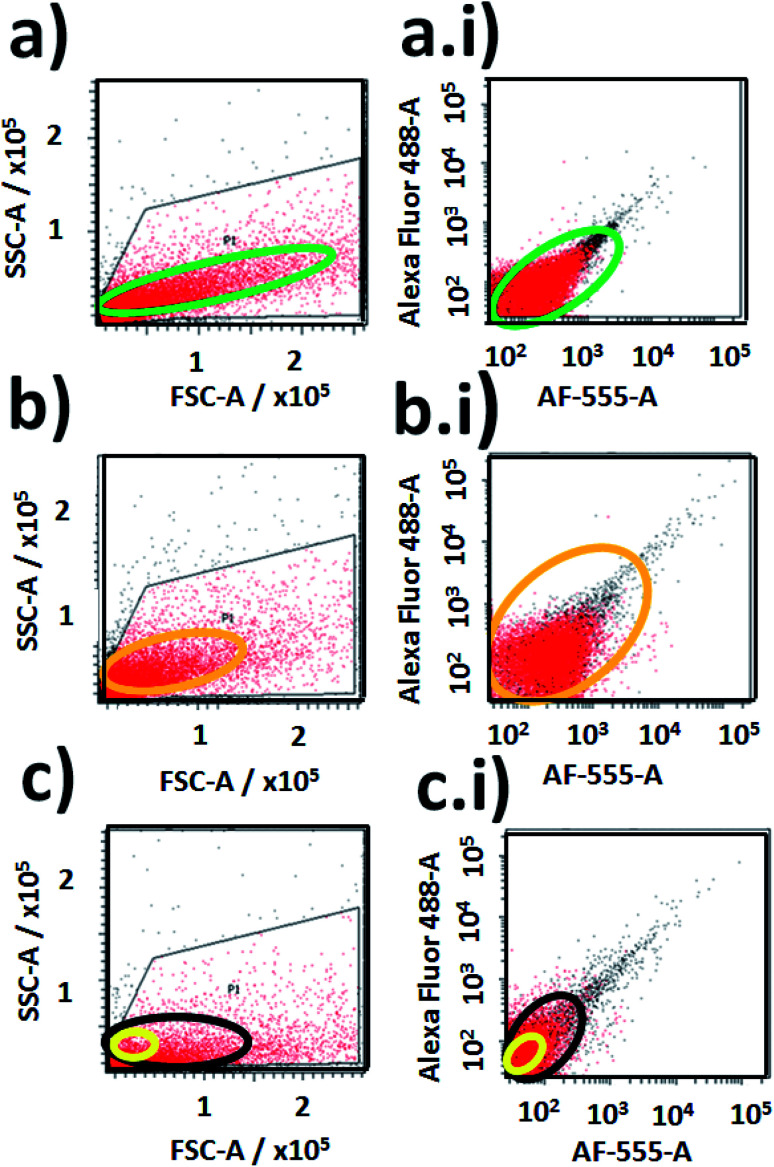
Fluorescence event detection *via* in-flow cytometry of labelled and non-labelled cyanobacteria: (a) and (a.i) correlation plots of SSC-A *vs.* FSC-A and a graph of fluorescent event counting between Alexa-Fluor 488-A *vs.* Alexa-F555-A from labelled cyanobacteria with multi-coloured NPs–(SiO_2_–RhB–Fl) nanoparticles; (b) and (b.i) correlation plots of SSC-A *vs.* FSC-A and a graph of fluorescent event counting between Alexa-Fluor 488-A *vs.* Alexa-F555-A from labelled cyanobacteria with mono-coloured NPs–(SiO_2_–Fl) nanoparticles; (c) and (c.i) correlation plots of SSC-A *vs.* FSC-A and a graph of fluorescent event counting between Alexa-Fluor 488-A *vs.* Alexa-F555-A from non-labelled cyanobacteria. Note: free mono-coloured NPs–(SiO_2_–Fl) and multi-coloured NPs–(SiO_2_–RhB–Fl) nanolabellers were detected in a reduced sized area highlighted with a yellow oval within the highlighted black oval in (c) for non-labelled cyanobacteria.

Then was evaluated the application of the developed nano-biolabelling methodology by in-flow cytometry with laser excitation and fluorescence detection. In order to do that, variable distributions were recorded of SSC and FSC (SSC: Side Scattered Light; and FSC: Foward Scattered Light) from the different labelled cyanobacteria and non-labelled biostructures (Fig. 14). As is known, it should be mentioned that the SSC parameter is a measurement of mostly refracted and reflected light that occurs at any interface within a cell where there is a change in refractive index.^64^ The SSC is collected at approximately 90 degrees to the laser beam by a collection lens and then redirected by a beam splitter to the appropriate detector. In this manner, the SSC parameter is proportional to cell granularity or internal complexity and it registers cleaner fluorescent event detections from samples with diminished background signalling. Moreover, the FSC is a measurement of mostly diffracted light and it is detected just off the axis of the incident laser beam in the forward direction by a photodiode. FSC provides a suitable method of detecting particles greater than a given size independent of their fluorescence and is therefore often used in immune-phenotyping to trigger signal processing.^65^

In addition variable fluorescent event detection counts were collected from the different samples with standard fluorescence parameters of Alexa-Fluor-488-A (laser excitation at 488.0 nm with emission filter placed at 530/30 nm) and AF-555-A (laser excitation at 555.0 nm with emission filter at 585/42 nm) (Fig. 14).

For cyanobacteria labelled with NPs–(SiO_2_–RhB–Fl) and NPs–(SiO_2_–Fl) nanoparticles, different distributions of SSC values were recorded. For cyanobacteria labelled with NPs–(SiO_2_–RhB@Fl), SSC values were recorded up to ×10^5^ (Fig. 14a); while for those labelled with NPs–(SiO_2_–Fl), values of 1 × 10^5^ and higher were collected (Fig. 14b). In this manner was recorded a higher number of fluorescent event detection counts of cyanobacteria labelled with NPs–(SiO_2_–Fl) (Fig. 14b(i)) than with NPs–(SiO_2_–RhB–Fl) (Fig. 14a(i)). This fact was explained in terms of the enhanced bio-FRET pathway by the emission of the NPs–(SiO_2_–Fl) towards the higher quantum yielding photoreceptor of cyanobacteria previously discussed.

In addition, from the analysis of FSC was observed a higher number of detection values within a larger interval of FSC values for cyanobacteria labelled with NPs–(SiO_2_–RhB–Fl) (Fig. 14a), and lower FSC values within a shorter range of values for those labelled with NPs–(SiO_2_–Fl) (Fig. 14b). This fact was supported by the larger sizes of the deposited NPs–(SiO_2_–RhB–Fl) than NPs–(SiO_2_–Fl) nano-labellers.

For non-labelled cyanobacteria, the fluorescent event detection distributions as well as the detection event counting were different in comparison to the previously discussed labelled biostructures. Smaller detection surfaces were obtained from SSC *vs.* FSC plots (Fig. 14c), accompanied by a lower number of detection counts (Fig. 14c(i)) than for cyanobacteria labelled with NPs–(SiO_2_–RhB–Fl) (Fig. 14a, a(i)) and NPs–(SiO_2_–Fl) (Fig. 14b, b(i)). Moreover, it should be highlighted that the free mono-coloured NPs–(SiO_2_–Fl) and multi-coloured NPs–(SiO_2_–RhB–Fl) nano-labellers were detected in reduced sized areas within the distribution of non-labelled cyanobacteria (highlighted yellow oval in Fig. 14c).

In this manner it was possible to apply the nano-biolabellers for cyanobacteria detection and counting with a versatile in-flow technique such as cytometry. However, further experiments should be done to validate this methodology.

**Fig. 15:**
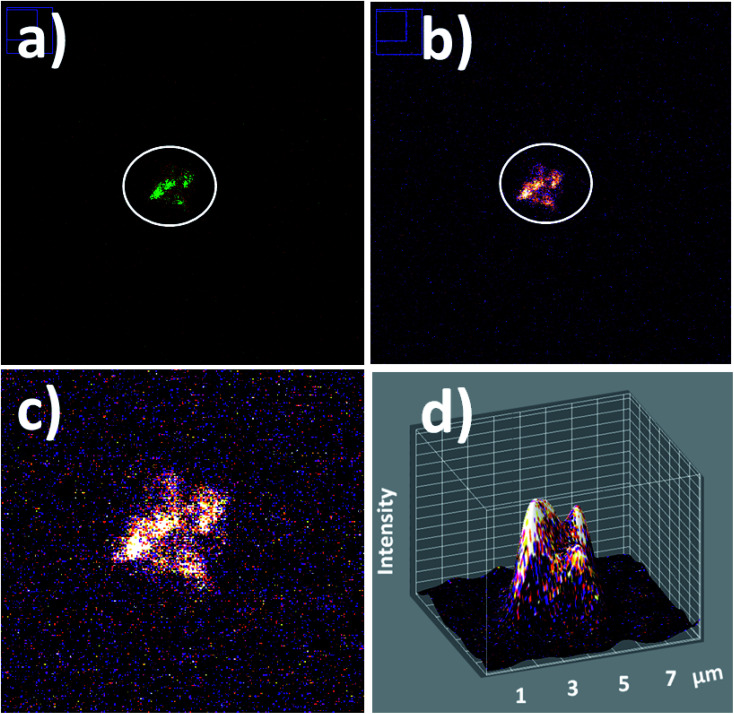
Laser fluorescence microscopy of individual small cyanobacteria aggregates: (a) red-green LUT; (b) fire LUT applied; (c) zoomed fire LUT image; and (d) 3D fluorescent surfaces of bacterial tetramers. Laser excitation at 488.0 nm.

In addition in order to evaluate applications by detection of these biostructures at low concentrations based on fluorescent bioimaging, colloidal dispersions were prepared with lower cyanobacteria concentrations. In this manner, by application of optimal NPs–(SiO_2_–Fl) nano-labellers, smaller bacterial aggregates were recorded (Fig. 15a and b) formed by dimers to tetramers of cyanobacteria (Fig. 15c). From these nano-biosurfaces, strong luminescent intensities were generated that permitted faster detection and counting with 3D fluorescence plots (Fig. 15d).

Finally it should be highlighted that using the developed methodology, individual ultraluminescent nano-biostructures were collected by in-flow cytometry as well as their detections by laser fluorescence microscopy. And at this point it should be mentioned that the non-classical light collected was from confined intermolecular interactions within silica nanoplatforms that permitted targeted light delivery *via* bio-FRET on biostructures that generated varied controlled emissions depending on the emitters incorporated within the nanostructures. In this way, these results showed potential applications of this methodology within confined microfluidics chips^66^ for biodetection and biophotonics applications.^67^

## 4. Conclusions

Variable silica nano-emitters were developed, formed *via* different fluorescent emitters with different quantum yields in order to tune their emission intensities based on FRET. From mono-coloured silica nanoparticles with the incorporation of Fl or RhB fluorescent dyes, strongly emitting 220.0 nm luminescent nanoparticles were recorded, with proportional quantum yields; while from multi-coloured silica nanoparticles, enhanced emissions were collected from 240.0 nm nanoparticles based on FRET. Increases were seen until 40% for multi-coloured silica nanoparticles.

These nanoparticles were evaluated for cyanobacteria labelling by non-covalent interactions and detected by laser fluorescence microscopy and in-flow cytometry. Thus, by TEM and laser fluorescence microscopy, the interactions were observed between the polar silica surfaces and polysaccharides naturally produced by the cyanobacteria that permitted targeted nanoparticle depositions.

By laser fluorescence microscopy, variable intensities from the luminescent nano-biostructures were recorded depending on the nano-labeller applied. Highly luminescent multi-coloured NPs–(SiO_2_–RhB–Fl) generated diminished emissions in comparison to NPs–(SiO_2_–Fl). This fact was explained by a bio-FRET coupling pathway with low quantum yields of natural chromophores from the biostructure. While, the application of NPs–(SiO_2_–Fl) nano-labellers produced higher emission by bio-FRET coupling with natural chromophores with higher quantum yields (known as photosystem I and II respectively).

Optimal biolabelling conditions were applied for in-flow cytometry experiments for cyanobacteria detection and counting. From cyanobacteria labelled with NPs–(SiO_2_–Fl) greater fluorescent event counts were recorded than with multi-coloured NPs–(SiO_2_–RhB–Fl) and NPs–(SiO_2_–RhB) nano-labellers. These results correlated with the results obtained *via* static fluorescence and laser fluorescence microscopy.

In this manner light delivery was achieved and controlled from confined fluorophores within silica nanoplatforms for biolabelling applications with optically active biomaterials. Finally, it should be mentioned that the enhanced emissions were recorded by a targeted bio-FRET pathway considering the intrinsic and natural chromophore compositions of the biostructures assayed. Thus, future applications to other types of biostructures were opened up based on light delivery from confined emitters.

## Conflicts of interest

There are no conflicts to declare.

## Supplementary Material
